# Lepra Vulgaris

**Published:** 1844-08-24

**Authors:** 


#### LEPRA VULGARIS.

Dr. J. C. Hall relates a case of long-continued lepra, attended with inflammatory symptoms and violent itching, in which, after premising venesection and the use of purgatives, he effected a cure by the cautious use of the liquor of the hydriodate of arsenic and mercury. He lays great stress upon the propriety of not exhibiting the arsenical preparation, until the indications of inflammatory action have been previously removed.—*Ibid.*

